# Study on the Optimal Groove Shape and Glue Material for Fiber Bragg Grating Measuring Bolts

**DOI:** 10.3390/s18061799

**Published:** 2018-06-01

**Authors:** Yiming Zhao, Nong Zhang, Guangyao Si, Xuehua Li

**Affiliations:** 1 Key Laboratory of Deep Coal Resource Mining of the Ministry of Education, School of Mines, China University of Mining and Technology, Xuzhou 221116, China; zhaoyiming@cumt.edu.cn (Y.Z.); Lixuehua@cumt.edu.cn (X.L.); 2 Shenzhen Oceanpower Co. Ltd., Shenzhen 518040, China; 3 School of Minerals and Energy Resources Engineering, University of New South Wales, Sydney, NSW 2052, Australia

**Keywords:** underground engineering, groove shape, glue material, FBG measuring bolts

## Abstract

Fiber Bragg grating (FBG) measuring bolts, as a useful tool to evaluate the behaviors of steel bolts in underground engineering, can be manufactured by gluing the FBG sensors inside the grooves, which are usually symmetrical cuts along the steel bolt rod. The selection of the cut shape and the glue types could perceivably affect the final supporting strength of the bolts. Unfortunately, the impact of cut shape and glue type on bolting strength is not yet clear. In this study, based on direct tension tests, full tensile load–displacement curves of rock bolts with different groove shapes were obtained and analyzed. The effects of groove shape on the bolt strength were discussed, and the stress redistribution in the cross-section of a rock bolt with different grooves was simulated using ANSYS. The results indicated that the trapezoidal groove is best for manufacturing the FBG bolt due to its low reduction of supporting strength. Four types of glues commonly used for the FBG sensors were assessed by conducting tensile tests on the mechanical testing and simulation system and the static and dynamic optical interrogators system. Using linear regression analysis, the relationship between the reflected wavelength of FBG sensors and tensile load was obtained. Practical recommendations for glue selection in engineering practice are also provided.

## 1. Introduction

Bolt supporting technology has been widely used in a variety of geotechnical engineering applications [1,2,3,4]. The axial stress distribution and force transfer mechanism of bolts have been studied by many researchers. To obtain the axial stress distribution and the force transfer mechanism of the bolt, various measurement techniques have been developed, such as strain gauges [5,6,7], vibrating-wire sensors [8], dynamometer [9], and non-destructive testing technology [10,11,12]. However, these techniques can only provide information about the short-term loading process due to environmental disturbances, corrosion, and electromagnetic interferences [13].

Given its unique high accuracy, excellent electromagnetic immunity, durability, and distributed measurement characteristics, the fiber Bragg grating (FBG) sensor has been widely applied as a perceived element and transmission medium to study the behavior of rock bolts or anchors by many studies in the field of underground engineering [14]. Each type of FBG sensor has the ability of monitor a specific reflected wavelength induced by strain deformation to calculate the load stress in the rock bolt or anchor. Schmidt-Hattenberge et al. [15] manufactured a glass fiber-reinforced polymer rock bolt for geotechnical strain measurements, whereby a Bragg grating was attached to the one mm wide and two mm deep groove of rock bolts by a special two-component epoxy. Lin et al. [16] bonded FBG in the 5 cm long, 0.3 cm wide, and 0.1 cm deep slot of the anchor end and on the surface of a single wire of the cable using “102” glue to obtain the relationship between the FBG wavelength shifts and stress. Chai el al. [17] glued three FBG sensors in the 1 mm deep and 30 mm long groove of a bolt, and used them to record axial stresses and strains along the bolt during a pull-out test. Weng el al. [18] adhered the FBG sensor to the surface of a tie-solder rod with “502” glue and wrapped with four layers of thermal plastic pipe, then monitored the seven strain conditions of the rock bolts during construction. Zhu et al. [19] captured the variations in internal displacement profiles in a model dam using sensing bars, in which FBG sensors were adhered in its grooves and covered with epoxy resin. Schroeck et al. [20] proposed a unique arrangement of FBG sensors to allow up to 20% strain measurements of steel rock bolts. Huang et al. [21] designed a novel distributed self-sensing fiber reinforced polymer (FRP) anchor rod with a built-in optical fiber sensor to predict the mechanical behavior of the anchor rod. Building upon FBG sensors, De Waele et al. [22] developed a load cell to measure the forces in the ground anchors of a quay wall, and concluded that fiber-based instrumentation is highly suitable for long-term monitoring purposes. Kim et al. [23] and Do et al. [24] encapsulated FBG sensors into the central king cable of a 7-wire steel strand to monitor the changes in tensile force and its distribution along the tendons during in-service state. They stated the sensors were embedded into the steel tube by a liquid glue with low viscosity. Xu et al. [25] measured the strain in 100 cm intervals along an anchor rock bolt grouted in the slope of intact rock using FBG strain sensors. Chen et al. [26] proposed five different FBG strain sensors and highlighted the prospective use of FBG in the strain monitoring of cables.

For a steel bolt, one common approach for installing the FBG sensors is to glue them inside the symmetrical grooves along the bolt rod, and then refill the groove using silica gel to protect the FBG sensors. Then, the FBG measuring bolt is manufactured for field applications to obtain the stress distribution and its variation along the bolt. However, the depth and shape of grooves cut within the bolt undoubtedly reduce the strength of the bolt itself, which may have a direct impact on the long-term monitoring accuracy of FBG sensors, especially given the complexity of the underground environment. In addition, the application of various types of glue material may also affect the monitoring accuracy of the FBG measuring bolt. Unfortunately, in the abovementioned literature, all factors interfering with the FBG monitoring sensors have not yet been properly investigated.

In this paper, the effects of groove shape on bolt strength were analyzed based on tensile experiments on conventional rock bolts. Following that, the stress distribution in an anchor bolt with different groove shapes was numerically analyzed by ANSYS, a continuum mechanics simulator. Furthermore, four commonly used glues and their effects on FGB monitoring sensors were assessed. Based on these results, the optimal groove shape and glue material are proposed to improve the monitoring performance of FGB bolts in underground engineering. Note that although the main engineering background in this research aims at understanding the impact of groove shape and glue material on FBG measuring bolts, the findings are also transferable to other strain gauge-based measuring bolts that also require cutting grooves and glue material.

## 2. Tensile Tests on Bolts with Different Groove Shapes

### 2.1. Tensile Test Conditions

**Figure 1:**
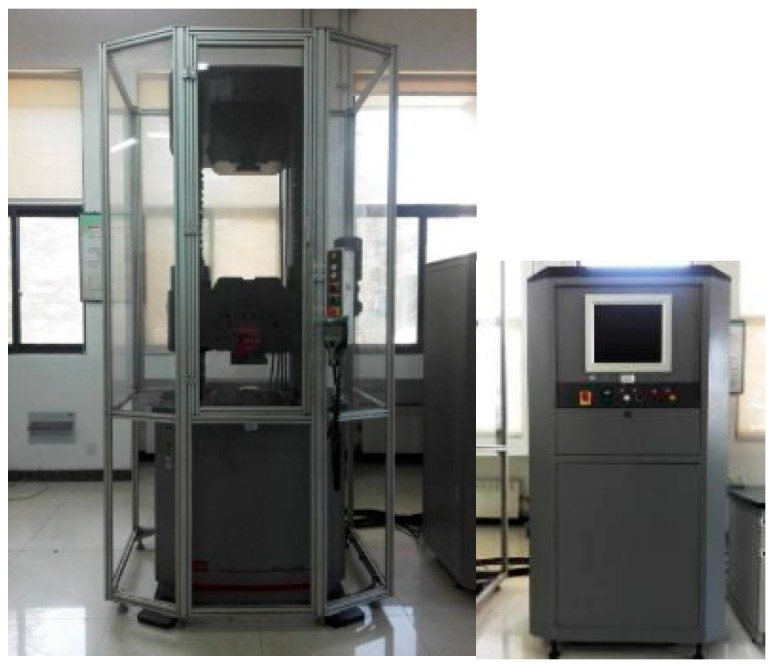
Mechanical testing and simulation (MTS) electrohydraulic servo testing machine.

The tensile tests were performed by using the mechanical testing and simulation (MTS) electrohydraulic servo testing machines, as shown in Figure 1. The basic parameters for the MTS machine were as follows: (1) peak load 1000 kN; (2) maximum tensile space 790 mm; (3) and displacement rate 0.5–90 mm/min.

**Figure 2:**
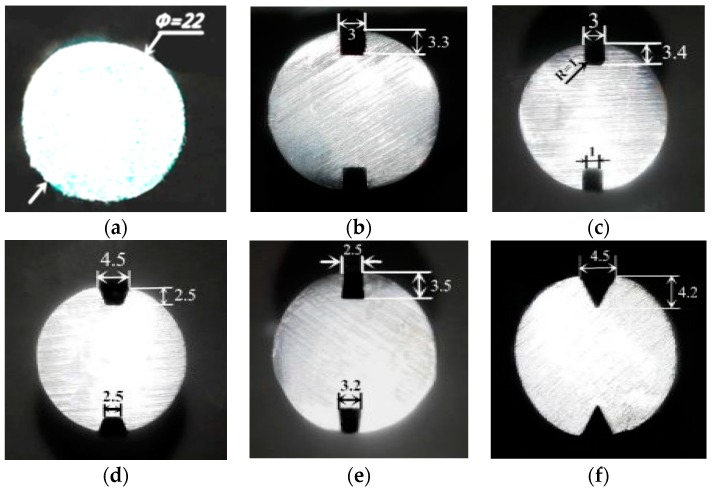
Comparisons between different groove shapes and sizes (mm): (**a**) no-groove; (**b**) U-groove; (**c**) U-groove with chamfer; (**d**) inverted trapezoidal groove; (**e**) trapezoidal groove; and (**f**) V-groove.

All specimens were cut from steel bolts that were used in underground coal mines. The length of the specimens was 250 mm, and the diameter was 22 mm. The specimens were classified into six categories based on groove shape. To rule out the size effect of various grooves, the six specimens aimed to achieve roughly the same cutting area for different shapes with a laser-cutting machine. Two independent specimens for each type of groove shape were manufactured and numbered to reduce sampling bias. The groove shapes and groove sizes for the specimens are shown in Figure 2.

### 2.2. Test Procedure and Results Analysis

**Figure 3:**
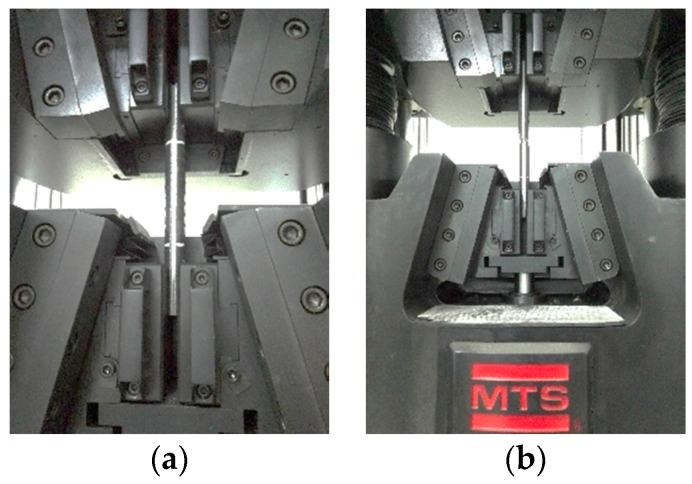
Tensile test procedure for the specimen: (**a**) before loading and (**b**) after loading.

The tensile test process used closed-loop control positioning. The tensile tests for the specimens were initiated at a constant loading rate of 5.1 mm/min under normal temperature and pressure conditions. The stress–strain responses of the specimens were recorded during the tensile loading process, and the frequency of data collection was 1.0 Hz. Note that the actual loading length of 110 mm was determined by deducting the length of the two ends held by the machine. The specimens were loaded until the specimen broke into two pieces. The images of the tensile tests are shown in Figure 3.

**Figure 4:**
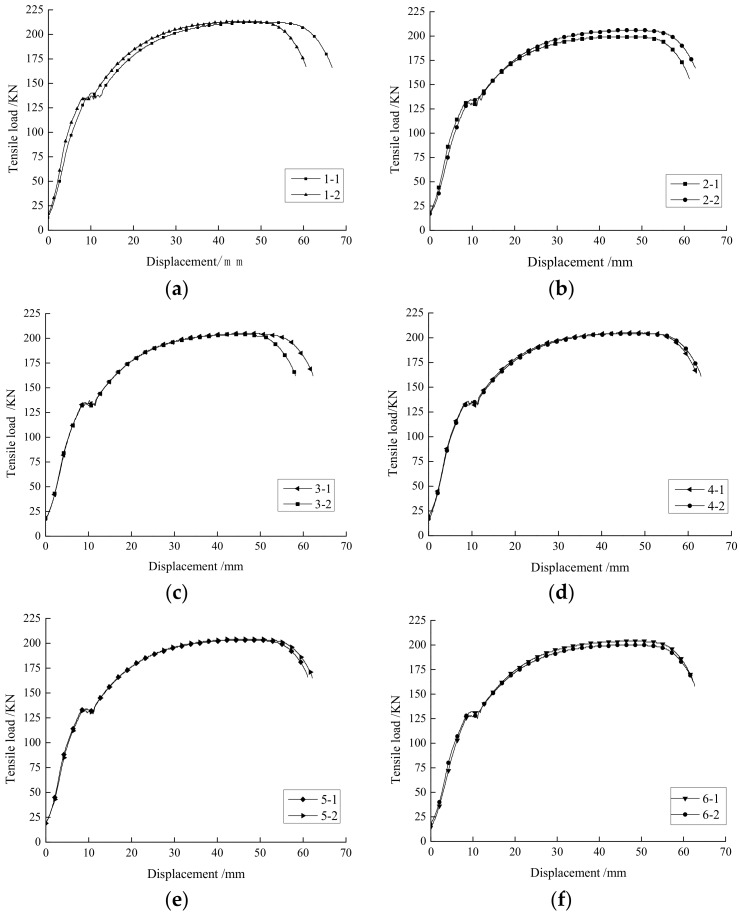
Relationship curves of tensile load-displacement for specimens: (**a**) no-groove; (**b**) U-groove; (**c**) U-groove with chamfer; (**d**) inverted trapezoidal groove; (**e**) trapezoidal groove; and (**f**) V-groove.

The tension strength and displacement relationships for all 12 tested specimens are shown in Figure 4.

The yield strength, ultimate strength, and failure strength for these specimens with different groove shapes are summarized in Table 1. We concluded that the creation of grooves can somewhat reduce the strength of specimens, and the effect of strength reduction varies with groove shape.

Figure 4 suggests that all 12 specimens followed almost the same trend in the tensile loading test. A linear elastic stage occurred between the tensile load and displacement during the initial stage of the tensile test. The specimens then reached the yield stage where the average yield strength of the no-groove specimens was 140 kN. In comparison with the no-groove type, the average yield strength for the specimens with the trapezoidal groove shape was 136 kN, with a strength reduction of 2.85%. Furthermore, the percentage of yield strength reduction for the V-groove specimen was the largest at 7.14%.

After the tensile load passed yield strength, the tensile load continuously increased, which resulted in the realignment of the internal grain structure of the specimens. This widely observed strain hardening behavior reflected the decrease in the specimen’s ability to resist further deformation despite the increasing tensile strength. After a period of yielding, the tensile load reached the peak strength and the internal structure of the specimen broke at around 80% of peak strength, which terminated the experiment. The average peak strength of the no-groove specimen was 213.5 kN. In comparison, the average peak strength dropped by 4.22% for both the U-groove with a chamfer and the trapezoidal groove, which was the lowest among all specimens with grooves. More than a 5% reduction in peak strength was observed in U-groove. By the time tensile load exceeded the peak strength, the lateral size of the specimen shrank dramatically. This necking phenomenon occurred shortly after the specimen broke. The breaking tensile load of the no-groove specimen was 166.5 kN, whereas it was nearly 8% lower for U-groove specimen.

The experimental results suggest that bolts with a trapezoidal groove have the least strength deterioration compared with the other groove shapes, which should be recommended for the manufacturing of FBG-based monitoring bolts.

## 3. Numerical Analysis of Stress Distribution of Bonded Bolts with Different Groove Shapes

### 3.1. Model Geometry and Boundary Condition

**Figure 5:**
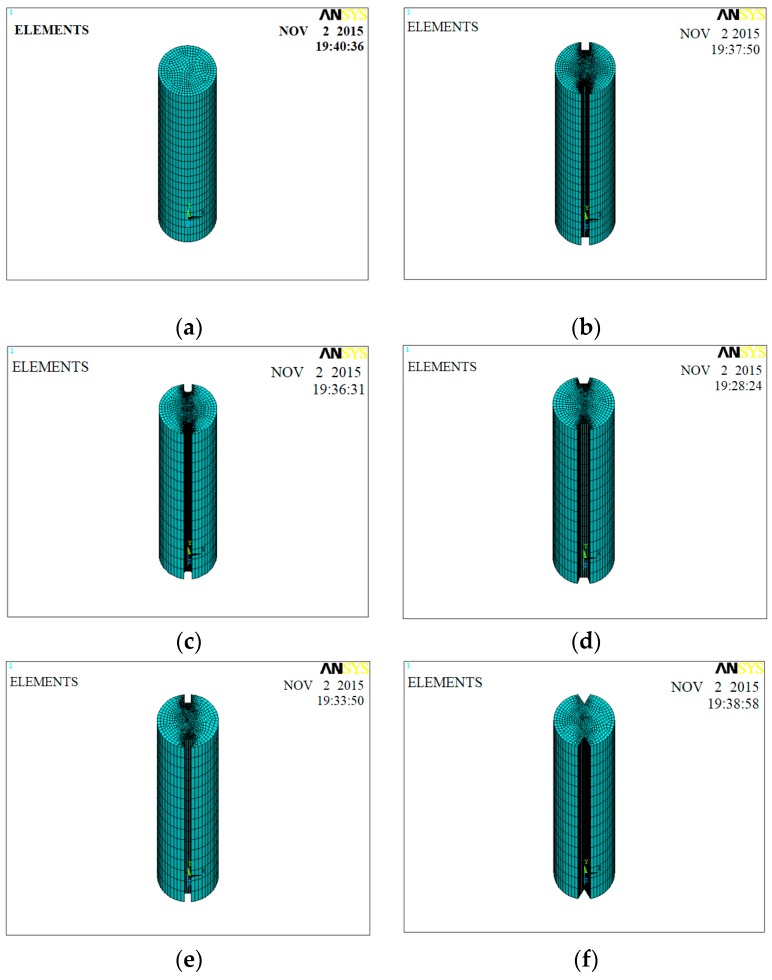
Model geometries for various groove shapes: (**a**) no-groove; (**b**) U-groove; (**c**) U-groove with chamfer; (**d**) inverted trapezoidal groove; (**e**) trapezoidal groove; and (**f**) V-groove.

To understand the stress distribution in bonded bolts with different groove shapes under tensile loading conditions, the following numerical models were developed in ANSYS. Note that numerical models developed in this section aimed to reproduce fully loaded bolts at actual working conditions, which are bonded to the surrounding rock, rather than a free state boundary, as shown in the tensile tests above. The bolt model was developed using 7400 SOLID, 95 elements, and 32,611 nodes. The material parameters used for this study were: model length 110 mm, diameter 22 mm, Young’s modulus for the bolting steel 20 GPa, and Poisson’s ratio 0.3. The boundary conditions of the bonded bolt were as follows: the x and z displacement were restrained; a fully fixed bottom surface; and the groove and top surfaces were free. The external stress applied to the model top surface was 119 kN. Model geometries (as shown in Figure 5) for various groove shapes and dimensions exactly followed the experimental designs as introduced in Section 2.1.

### 3.2. Simulation Results 

**Figure 6:**
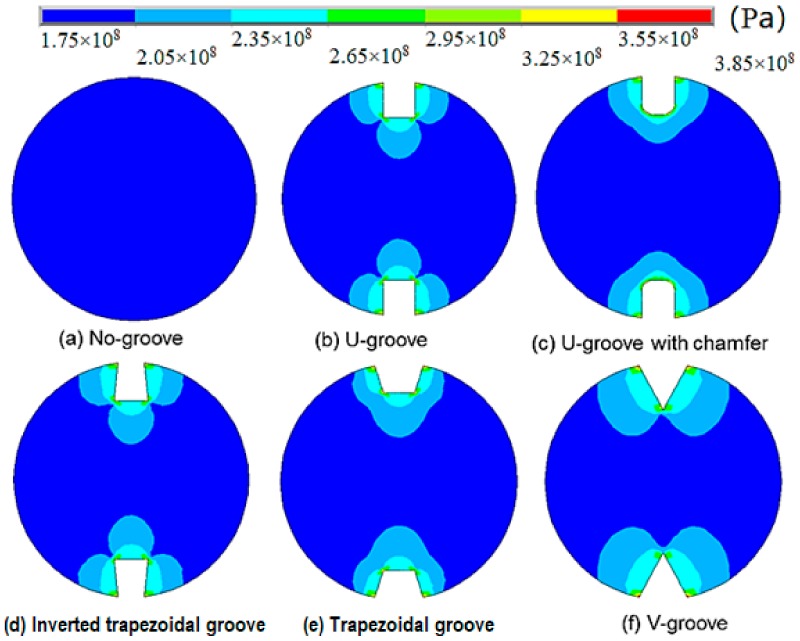
Stress distribution in the central cross-section for bonded bolts with different groove shapes.

The stress contours of the middle cross-section for different groove shapes are shown in Figure 6. After introducing grooves, stress concentration occurred surrounding each groove. For the V-shaped grooves, which had the largest stress disturbance area, the magnitude of the elevated stress can be as twice as much as the stress in the undisturbed area. The majority of elevated stress in the disturbed zone ranged from 1 to 1.3 times the undisturbed stress. Notably, the stressed measured by FBG (or any other type strain-gauge) is not the true stress state in a no-groove bolt, but an elevated stress state induced by the specific shape of the grooves. Due to this artificial effect, compared with no-groove bolts, the measured stress in grooved bolts can be amplified by a factor of 1.3, as shown in Figure 6. This needs to be taken into consideration in engineering practice.

The maximum stress values in the central cross-section for different groove shapes are summarized in Table 2. The maximum stress value for the no-groove bolt was 179 MPa. Under the same tensile load, the maximum stress value for the V-groove bolt was 378 MPa and the stress concentration factor was 2.11 by comparing with the no-groove case, which is the highest value compared with the other types of grooved bolts. Correspondingly, the maximum stress value for the bolt with the trapezoidal groove was 335 MPa and the stress concentration factor was 1.87, which is the smallest among all scenarios.

The modelling results are consistent with the experiment findings. The creation of a trapezoidal groove has the least impact on the bolt strength, since the stress concentration at the corner points are the lowest. Conversely, the V-groove, which may induce twice as much stress concentration, should be avoided in engineering practice.

## 4. Glue Material Recommendation

### 4.1. Glue Materials

FBG sensors are normally glued inside the symmetrical grooves along the bolt axial direction during the manufacturing of FBG measuring bolts. The glue material can not only ensure FBG sensors remain inside the cuts, but also be able to deform in conjunction with the deformation of the bolts. Four typical glues were used to attach FBG sensors to specimens in this study. Table 3 summarizes general information about the four type of glues we used in this research. Note that all specimens studied in this section were applied with the trapezoidal groove, since it had the least impact on bolt strength, as found in previous sections.

**Figure 7:**
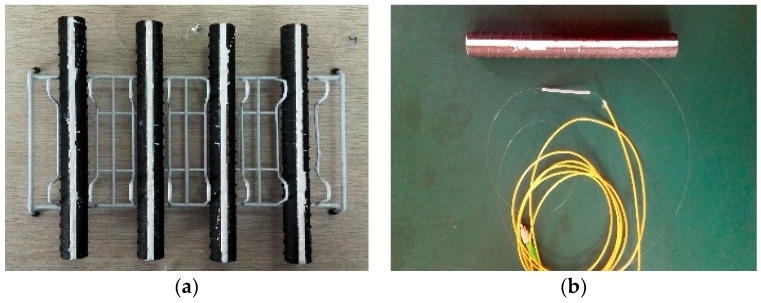
Photos of packaged specimens: (**a**) specimens using different glues and (**b**) specimen with jumper connection.

Since FBG sensors are made of glass material, to ensure their integrity, the gluing process should follow the proposed manufacturing steps during the preparation of the specimens. First, sandpaper was used to grind each groove until its bottom surface was sufficiently smooth. Second, acetone and alcohol were used to scrub the groove to remove dust and impurities. After that, the glue was spread into the grooves while maintaining the bare FBG sensors in a straight-line orientation, which was followed by placing the specimen in a fixture to allow the glue to solidify. The required curing time at 25 °C was 2 h, for 60 °C was 1 h, for 80 °C was 30 min, and for 100 °C was 5 min. The sample was then cooled down to room temperature (about 19 °C). Finally, a protection plastic thin tube was placed on the optical fiber except the optical grating part, and the grooves were filled with 704 silica to encapsulate and protect FBG sensors. The 704 silica is a single-component adhesive material with good adhesion, high strength, and no corrosion, which has excellent electrical insulation, sealing performance, moisture resistance, and anti-seismic and aging resistance. Note that the characteristics of FBGs were as follows: FBG length 10 mm, fiber type was SMF-28C fiber, FWHM BW ≤ 0.3 nm, reflectivity ≥ 85%, acrylate recoat, and FC/APC connector. The completely packaged specimens with jumper connections are shown in Figure 7.

### 4.2. Tensile Test and Data Analysis

**Figure 8:**
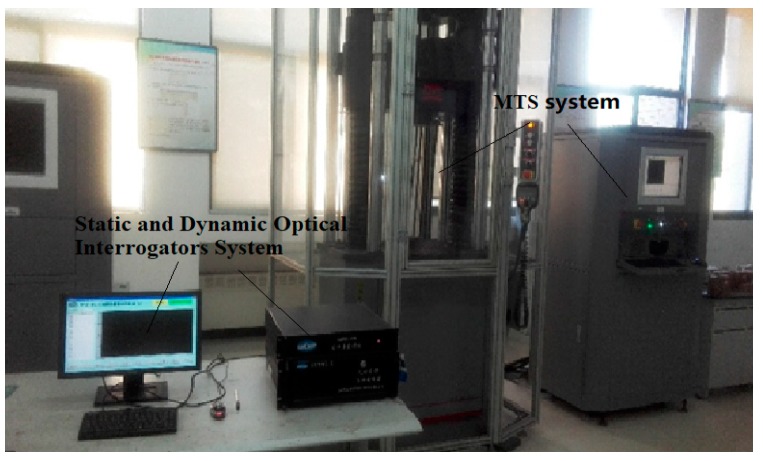
The mechanical testing and simulation (MTS) system and static and dynamic optical interrogators (SDOI) system used for the ensile loading experiment.

The tensile test of the packaged specimens was performed using an MTS system and static and dynamic optical interrogators (SDOI) system. The SDOI system is composed of the static and dynamic optical interrogators, an industrial personal computer, and corresponding software, which can be used to record the specific reflected wavelength of FBG sensors embedded in a bolt with a trapezoidal groove. The general information about the interrogator is shown in Table 4. An MTS system is used to record the tensile loading exerting on the packaged specimens. During the tensile loading process, the reflected wavelength of the FBG sensors changes accordingly. Then, the tensile load and the reflected wavelength of the FBG sensor were recorded during the tensile process, with a 1.0 Hz frequency of data collection. The test system is shown in Figure 8.

**Figure 9:**
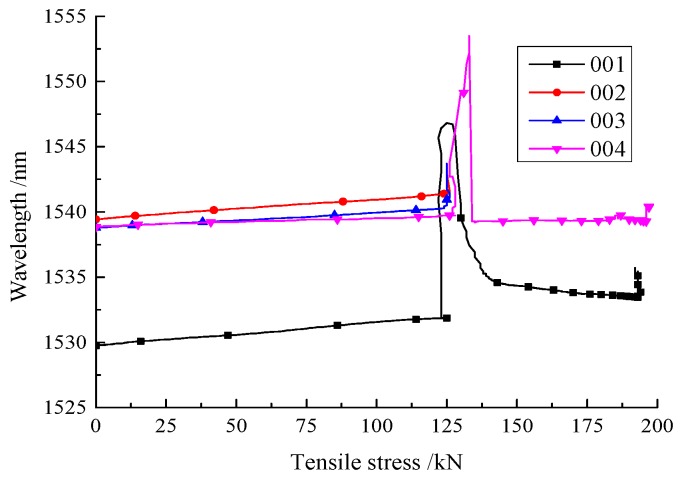
Wavelength variation during tensile loading.

The relationship curves between the tensile loads and the reflected wavelength of the FBG sensors with different glues are shown in Figure 9. Peak wavelength was calculated from the reflection spectrum using a built-in software package in the interrogator purchased for this project. The software can automatically search for the peak wavelength once the reflection spectrum was obtained. Before the bolts reached the yield strength, the reflected wavelength of the FBG sensors for all specimens exhibited a reliable linear relationship with the increase in tensile load. However, when the tensile load exceeded yield strength, the monitoring of the reflected wavelength of specimens No. 002 and No. 003 was interpreted and started recording zero. This indicates that the FBG sensors, attached by the 502 glue and the epoxy resin AB, might have detached from the bolt or were partially damaged. Although the reflected wavelength of specimens No. 001 and No. 004 were still recording positive data, they both experienced dramatic fluctuation, and their relationship with tensile load was no longer linear, which means the FBGs either broadened or split. The reflected wavelengths for these two specimens fell to zero when the tensile load exceeded the peak strength, which suggested the deterioration of the last two specimens. Correspondingly, based on this result, the evolution of the reflected wavelength can be used to examine the damage to the bolt [27,28] if the perfect gluing quality of FBGs is achieved.

From the above measurement results, when the tensile load on the bolt exceeded its yield strength, the bolt was expected to experience a significant amount of tensile displacement. Since FBG sensors have relativity limited ability to resist tension deformation, this non-compatible deformation that occurred between the bolt and the FBG sensor may lead to the malfunction of these monitoring sensors. Therefore, FBG sensors can provide accurate results only if the recorded load range is within the yielding strength of the bolt being monitored. However, the malfunction of FBG sensors can be seen as an indicator that bolts have serious internal damage, which may be used in engineering to diagnose bolt integrity.

**Figure 10:**
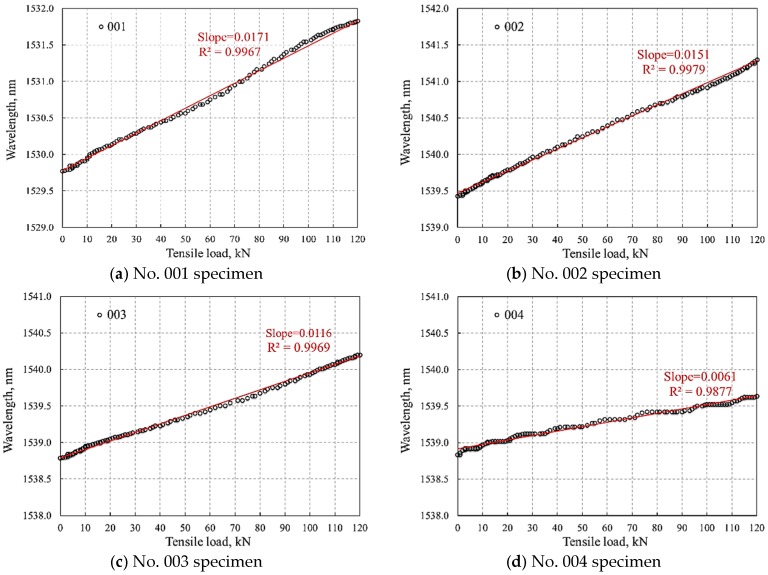
Linear relationship between reflected wavelength and tensile load in specimens with different glues.

The accuracy of using FBG-based sensors to measure tensile load on bolts is highly reliant on the linear response of the reflected wavelength to tensile deformation, which was only observed in the elastic stage before reaching the yielding point. Therefore, for the purpose of bolt tensile load monitoring, a sound and robust linear relationship between tensile load and reflected wavelength is critically important in FBG-based monitoring of bolts. The elastic range of the four tested specimens during their tensile tests were linearly fitted using regression method as shown in Figure 10.

Although a relatively high correlation (*R*^2^ > 9.87) was obtained for all four specimens, the selection of glue material had various impacts on the wavelength–tensile load relationship. The specimens No. 001–No. 003 all presented a constant and reliable linear relationship with a correlation coefficient over 0.995. Conversely, the linear curve of specimen No. 004, which used H814-epoxy resin glue, fluctuated over its elastic period, showing the lowest correlation. In addition, the linear slope of the reflected wavelength using this glue was the smallest among all samples, which indicates the measured tensile load would be much more sensitive to wavelength change compared with the other three. A tiny wavelength change may result in a large variance in measured tensile load. Therefore, in engineering practice, we recommend selecting glues with robust linearity and proper sensitivity response to the reflected wavelength, for instance, glue types No. 001 and No. 002 in this research.

The effect of different glues on FBG bolts is mainly determined by glue components and the proportion of each component. For the modified acrylate glue, its main components are methacrylate with a mix of toughening agents, reinforcing agents, and stabilizers. Compared with others, this type of glue can more firmly attach FBG sensors to bolts, which satisfies the harmonic deformation between FBG sensors and bolts before reaching yield strength.

Note that due to the limited amount of samples being tested in this research, the variability of sample performance was not fully considered. Although not directly related with this research topic, the four glues tested here were also used in other research projects involving FBG bolts led by the authors and all glues showed a relative consistent performance. Nevertheless, repeatability of the gluing procedure may have an influence on the testing results, and this will be investigated in the future work.

## 5. Conclusions

This research focused on investigating the effect of various groove shapes on the reduction of bolting strength when using FBG measuring bolts. The performance of five different groove types were assessed in both tensile load tests and numerical simulations. Both experimental and numerical results suggested that the trapezoidal groove has the least impact on bolting strength, with only a 0.37% reduction in tensile yield strength. Another interesting finding from the numerical modelling results is the FBG (or any other type strain-gauge) measured stress is not the true stress state in a no-groove bolt, but an elevated stress state induced by the specific shape of grooves. Four different types of glues normally used in practice were also tested to further understand their role in affecting FBG measuring bolts, according to the test results, we recommend that, among four tested glues, the modified acrylate glue is the optimal glue material for manufacturing the FBG measuring bolt.

## Figures and Tables

**Table 1:** Strength of specimens with different groove sharps.

Groove Shape	No Groove		U-Groove		U-Groove with Chamfer		Inverted Trapezoidal Groove		Trapezoidal Groove		V-Groove	
Specimen No.	1–1	1–2	2–1	2–2	3–1	3–2	4–1	4–2	5–1	5–2	6–1	6–2
Yield strength (kN)	140	140	131	135	135	134	134	132	136	136	132	128
Average yield strength (kN)	140		133		134.5		133		136		130	
Reduction (%)	-		5.00		3.93		5.00		2.85		7.14	
Peak strength (kN)	215	212	199	206	205	204	203	204	205	204	204	200
Average peak strength (kN)	213.5		202.5		204.5		203.5		204.5		202	
Reduction (%)	-		5.15		4.22		4.68		4.22		5.39	
Failure strength (kN)	172	166	156	151	162	162	166	165	164	165	164	158
Average failure strength (kN)	166.5		153.5		162		162.5		165.5		161	
Reduction (%)	-		7.81		2.70		0.60		0.60		3.30	

**Table 2:** Maximum stress values in the central cross-section of a bonded bolt.

Groove Type	Maximum Stress Value (MPa)	Stress Concentration Factor
No-groove	179	-
U-groove	359	2.01
U-groove with chamfer	348	1.94
Inverted trapezoidal groove	365	2.04
Trapezoidal groove	335	1.87
V-groove	378	2.11

**Table 3:** General information of four type of glues used in this research.

No.	Groove Shape	Glue Name	Main Component
001	Trapezoidal groove	Modified acrylate glue	Methacrylate and mix of some toughening agent, reinforcing agent, and stabilizer
002	Trapezoidal groove	502 glue	Ethyl α-cyanoacrylate and mix of some toughening agent, reinforcing agent, and stabilizer
003	Trapezoidal groove	Epoxy resin A-B glue	Two components based on epoxy resin with high temperature resistance
004	Trapezoidal groove	H814-Epoxy resin glue	Epoxy resin, curing agent, methyl tetrahydrophthalic anhydride, aluminum hydroxide, and polyester poly

**Table 4:** General specifications of the interrogator used in this study.

Part	Parameter
Optical channels	4 individual channels
Wavelength range	1525–1565 nm
Minimum wavelength spacing	0.4 nm
Wavelength precision	±1 pm
Scan and report rate	500 Hz
Optical connector	Ferrule Connector/A-Physical Connection
Power supply	220V AC 1A
Communication	USB 2.0
Operating temperature	0–40 °C
Dimensions	480 × 400 × 95 mm
