# Erratum: Zhao, Y.; Zhang, N.; Si, G.; Li, X. Study on the Optimal Groove Shape and Glue Material for Fiber Bragg Grating Measuring Bolts. *Sensors* 2018, *18*, 1799

**DOI:** 10.3390/s19071723

**Published:** 2019-04-10

**Authors:** Yiming Zhao, Nong Zhang, Guangyao Si, Xuehua Li

**Affiliations:** 1 Key Laboratory of Deep Coal Resource Mining of the Ministry of Education, School of Mines, China University of Mining and Technology, Xuzhou 221116, China; zhaoyiming@cumt.edu.cn (Y.Z.); Lixuehua@cumt.edu.cn (X.L.); 2 Shenzhen Oceanpower Co. Ltd., Shenzhen 518040, China; 3 School of Minerals and Energy Resources Engineering, University of New South Wales, Sydney, NSW 2052, Australia; 4 State Key Laboratory of Coal Resources and Safe Mining, China University of Mining and Technology, Xuzhou 221116, China

The authors wish to correct the affiliation of co-author Guangyao Si, due to name changes of which he was unaware during his leave of absence. The correct affiliation is 3. School of Minerals and Energy Resources Engineering, University of New South Wales, Sydney, NSW 2052, Australia and 4. State Key Laboratory of Coal Resources and Safe Mining, China University of Mining and Technology, Xuzhou 221116, China. The authors would like to apologize for any inconvenience caused to the readers by these changes.
